# Urinary extracellular vesicle as a potential biomarker of exercise-induced fatigue in young adult males

**DOI:** 10.1007/s00421-022-04995-3

**Published:** 2022-07-04

**Authors:** Suhong Park, Hyo Youl Moon

**Affiliations:** 1 Department of Physical Education, Seoul National University, Seoul, Republic of Korea; 2 Institute of Sport Science, Seoul National University, 71-1, 407, Gwanak-ro, Gwanak-gu, Seoul, 08826 Republic of Korea

**Keywords:** Urine, Extracellular vesicle, Fatigue, Exercise, miRNA

## Abstract

**Purpose:**

Previous studies have suggested that circulating extracellular vesicles (EVs) arise after high intensity exercise and urine could reflect the plasma proteome. Herein, we investigated the characteristic of urinary EVs from healthy young adult males who had completed a maximal effort exercise test.

**Methods:**

Thirteen healthy men completed a 20 m shuttle run test (20 m SRT). Fresh urine samples were collected at first morning, right after, and 1 h rest after 20 m SRT. Also, blood lactate, heart rate, rating of perceived exertion, and blood pressure were measured before, right after, and 1 h rest after 20 m SRT. Urinary EVs were analyzed using Exoview instrument and microRNAs (miRNAs) sequencing on urinary EVs were performed.

**Results:**

Urinary EVs increased significantly after exercise and returned to baseline value after 1 h of rest. miRNA sequencing on urinary EV revealed alterations in four miRNAs (1 up and 3 down) and nine miRNAs (2 up and 7 down) in pre- vs. post- and post- vs. post-1 h samples, respectively. Lastly, bioinformatic analysis of urinary EV miRNA suggests that predicted target genes could affect PI3K-Akt, mitogen-activated protein kinase, and insulin pathways by exercise.

**Conclusions:**

Exercise to voluntary exhaustion increased the number of EVs in urine. Also, miRNAs in urinary EVs were altered after exercise. These findings could indicate the possibility of using the urinary EVs as a novel biomarker of acute exercise-induced fatigue.

## Introduction

Participating in regular exercise is one of the best ways to prevent chronic diseases and maintain longevity. Even a single bout of exercise could be beneficial for cardiac function (Boutcher et al. 2011; Pober et al. 2004). Exercise could change the metabolic phenotype of our system (Amar et al. 2021; Frampton et al. 2021; Kim et al. 2019), including increased insulin sensitivity, glucose uptake (Hayashi et al. 2005), oxidative capacity, secretion of various peptides, extracellular vesicles, and metabolites (McGee and Hargreaves 2020). However, excessive exercise or training without sufficient recovery could evoke short-term overreached or overtraining syndrome, resulting in constant fatigue and underperformance (Halson and Jeukendrup 2004; Urhausen and Kindermann 2002).

The common parameters for quantifying the intensity of training are heart rate (HR), rating of perceived exertion (RPE), and blood lactate (BLa) (Wallace et al. 2014). However, these markers have limitations. RPE, which is a subjective marker, sometimes overestimates the intensity (Scherr et al. 2013). BLa analysis has hygiene issues, and it is difficult to collect blood samples several times (Green et al. 2006). Similarly, although HR is a useful marker, self-monitoring and self-regulating the exercise intensity with HR is difficult (Ciolac et al. 2015). Blood pressure (BP), which increases progressively with exercise intensity, could elevate during rest due to anxiety (Miyai et al. 2002). Therefore, there is a need for an alternative marker to objectively detect individually perceived intensity or fatigue. We consider EVs to be a resource that complements, not replaces, these fatigue markers.

EVs are found in most bodily fluids, such as blood, urine, and saliva (Colombo et al. 2014). Among them, urine is easy to collect in relatively large volumes compared to other bodily fluids, and not only contains kidney-derived proteins, peptides, and metabolites, but also reveals the components present in plasma (Harpole et al. 2016). These small sized vesicles are secreted by the cells (Jeppesen et al. 2019). Thus, EVs contain integral components, such as proteins, mRNA, and miRNA of originating cells (Yellon and Davidson 2014). In this regard, they could act as representatives of the cellular origin and physiological state of the secreting cells (Zhang et al. 2019).

The kinetics of EVs in plasma shows a similar increase as BLa during incremental cycling (Frühbeis et al. 2015a). High intensity endurance exercise was reported to elevate EVs in circulation (Wilhelm et al. 2016, 2017). Resistance training after endurance exercise also facilitates multivesicular body (MVB) biogenesis, partially stimulating exosome biogenesis (Garner et al. 2020). Studies have suggested that an acute bout of exercise can stimulate the release of EVs from muscles and other tissues (Safdar et al. 2016; Whitham et al. 2018). In addition, endurance exercise can change the proteomics of urine (Kohler et al. 2015). Therefore, it is possible that exercise could affect the characteristics of urinary EVs and their components.

However, no study has elucidated the effects of exercise on urinary EVs. Therefore, this study attempted to define the characteristics and possible functions of urinary EVs after the maximal effort test with two hypotheses. First, the number of EVs will be altered after 20 m SRT. Second, the profiles of miRNAs in urinary EVs will be changed after 20 m SRT.

## Materials and methods

### Ethical approval

All experimental procedures were approved by the Institutional Review Board of Seoul National University in accordance with the standards of the Declaration of Helsinki of the World Medical Association (IRB No. 2009/003-028). All subjects were informed about the procedures and purpose of the study by oral and written forms, and they confirmed to take part in it.

### Subjects

**Fig. 1:**
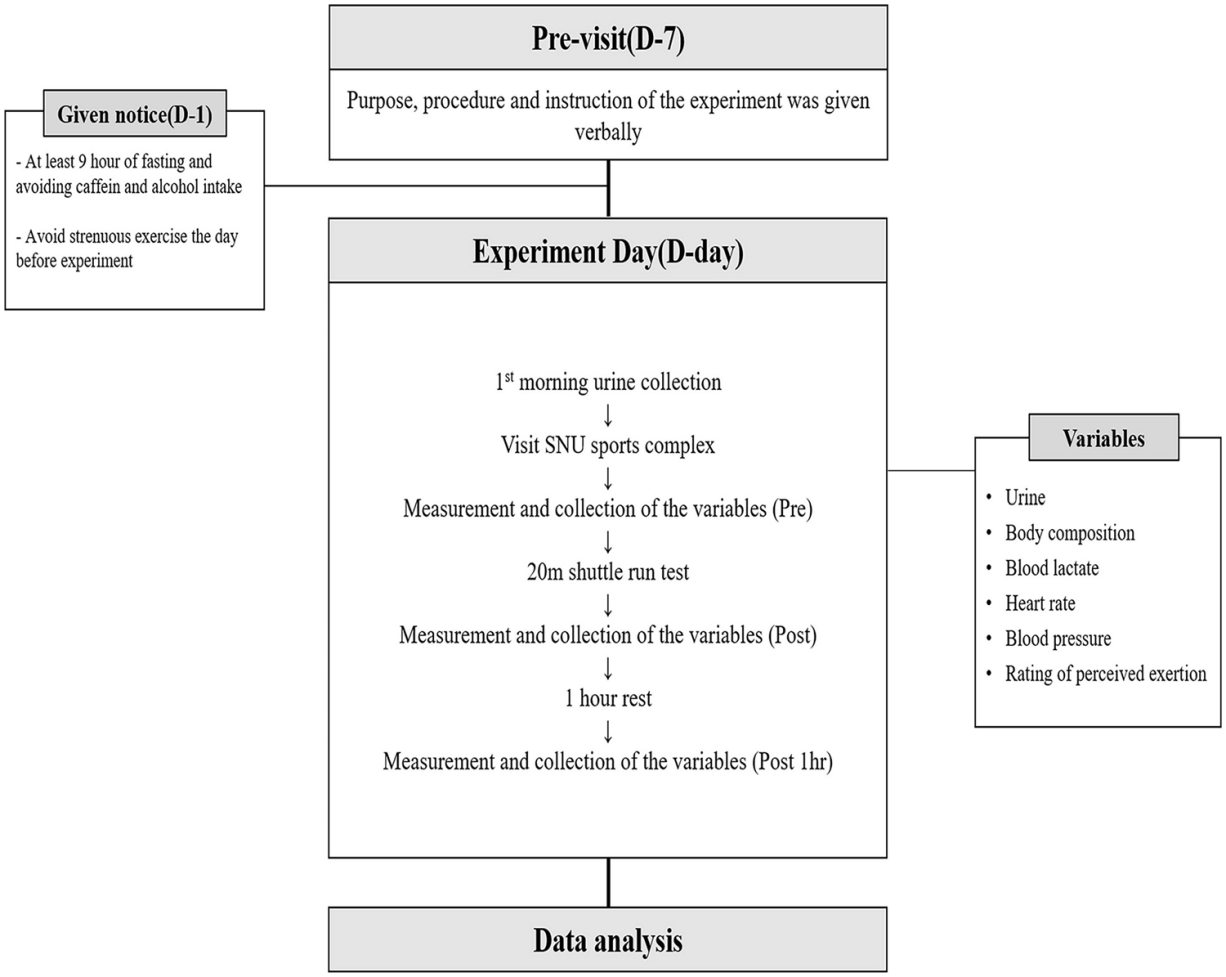
Experimental procedure

Thirteen healthy men aged 25.5 ± 2.0 years were recruited from Seoul National University in response to an advertisement. Men with a history of cardiovascular disease, musculoskeletal disease, or psychological disorder were excluded (more details about the inclusion and exclusion criteria are shown in Table 1). Subject characteristics are shown in Table 2. All participants were encouraged to fast for at least 9 h before the test day. They were also informed about the procedures of the study and the risks of 20 m SRT. Written consent was obtained from all participants before the experiment (Fig. 1).

**Table 1:** Inclusion and exclusion criteria

Inclusion criteria	Exclusion criteria
Subject who(se) Agreed to participate in this study and sign a written consent from Aged 20 to 30 years BMI between 18.5 kg/m^2^ and 30.0 kg/m^2^ Normal BP less than 140 mmHg (systolic) and BP less than 90 mmHg (diastolic) Body temperature less than 37.5 °C when examined	Subject who(se) BP is greater than 140 mmHg (systolic) or the relaxer BP is greater than 90 mmHg (diastolic) Had kidney disease BMI is less than or equal to 18.5 kg/m^2^ or greater than 30 kg/m^2^ Cannot walk or run Had hyperlipidemia, asthma, diabetes, bronchitis, anemia, thyroid disease, cardiovascular disease (hypertension, stroke, etc.), liver disease, musculoskeletal disorders, neurological disorders, or past history, or subscribed relate medications Had surgery within 6 months prior to his first visit Considered as inappropriate to participate in this study by a researcher

**Table 2:** Subject characteristics

Anthropometric characteristics	
Variables (*n* = 13)	
Age (year)	25.5 ± 2.0
Height (cm)	177.3 ± 5.6
Weight (kg)	72.0 ± 5.7
Fat percentage (%)	13.5 ± 4.6
Fat free mass (kg)	35.6 ± 3.2
20 m SRT result	
Repetition	81.2 ± 13.9

### Urine sample collection and preparation

Participants were asked to collect their first morning urine (Pre) into 50 mL sterile containers (SPL, Gyeonggi-do, Korea). Immediately after 20 m SRT (Post) and after 1 h (Post 1 h) of rest, urine samples were collected within 5 min. After collection, samples were filtered through a Minisart® 0.2 µm filter (Sartorius Stedim, Dourdan, France), and the filtered samples were frozen at − 80 °C for further analysis.

### Body composition and blood pressure measurement

Weight, fat percentage, and fat-free mass of participants were measured using an Inbody 720 (Biospace, Korea). Unnecessary outwear and accessories were removed before measurements. Brachial BP was measured in duplicate using a digital sphygmomanometer (Omron™, Matsusaka, Japan). BP was assessed on the non-dominant arm (in this study all left) to measure the rest state (rested 3 min on chair) (Pre), immediately after 20 m SRT (Post), and after 1 h of rest (Post 1 h).

### Measurement of rating of perceived exertion

The 15-point Borg rating scale of RPE (Borg 1970) which ranges from 6 to 20, was used to examine perceived exertion after 20 m SRT. Verbal instructions of the standard were given to the participants before measurements. RPE was obtained at the rest state before the test (Pre), immediately after 20 m SRT (Post), and after 1 h of rest (Post 1 h).

### EV isolation

EV fractions from filtered urine samples were isolated by using miRCURY Exosome Cell/Urine/CSF Kit (Qiagen, Germany) following the manufacturer’s instructions. In short, 4 mL of precipitation buffer B was added to 10 mL of the urine sample. All samples were incubated at 4 °C overnight (16 h) and centrifuged at 3200 × *g* for 30 min at 20 °C. The obtained pellet was resuspended in 50 µL of resuspension buffer and frozen at − 80 °C.

### Quantification of urine-derived EVs

The physical and biological properties of EVs in urine samples were characterized using ExoView R-100 and ExoView tetraspanin kit including anti-CD 81, anti-CD 63, and anti-CD 9 immobilized chips, labeling agents, washing solutions (solutions A and B), and blocking agents (Nanoview Bioscience, Boston, USA) (Gori et al. 2020).

First, 35 µL of the sample diluted 1:30 in distilled water was dropped onto the ExoView tetraspanin chip and incubated overnight at room temperature (r. t.). After the incubation process, the sample-loaded chip was washed thrice with 1 mL of solution A. Subsequently, the EVs on the chip were labeled using 250 µL of a mixture of anti-CD 81/AF 555, anti-CD 63/AF 647, and anti-CD 9/AF 488 and incubated for 1 h to analyze the colocalization of tetraspanin on the surface of the EV. In this case, the labeling antibody (Ab) was diluted in a mixture of solution A and blocking solution at 1:600. Finally, the chip was rinsed with 1 µL of solutions A and B and dried at r.t. The EV-captured chip was scanned using an ExoView R-100 (Nanoview Bioscience, Boston, USA).

### BLa measurement

Using Accu-Chek Softclix plus lancing device and Softclix (Roche®, Germany), 5 µL of blood from the fingertip was aspirated into an enzyme-coated electrode test strip (Arkray, Japan). Concentration levels of BLa were checked with a Lactate Pro2 (Arkray, Japan) device before (Pre), immediately after (Post), and after 1 h of the 20 m SRT (Post 1 h).

All equipment was cleaned and operated according to the manufacturer’s instructions. Blood samples were analyzed within 30 s of collection.

### 20 m shuttle run test

The participants participated in continuous running between two cones placed 20 m apart in response to the recorded beeps. Before carrying out the test, participants made sure they could hear the beep clearly from each end of the line. After 5 min of warm-up, subjects started running back and forth in response to the beep sound and counts recorded in Korean, with an initial speed of 8 km/h. The speed progressively increased in accordance with the test grade. The total shuttle run number was checked when participants could not reach the end line in response to the beep more than twice.

The test was conducted between 09:00 and 11:00 AM in the Seoul National University Sports Complex.

#### miRNA sequencing and bioinformatic analysis

EV RNAs obtained from three urinary EV samples were used to generate sequencing libraries with the SMARTer smRNA-Seq Kit for Illumina (Takara Bio, Shiga, Japan) according to the manufacturer’s protocol. In short, input RNA was at first polyadenylated to provide a priming sequence for an oligo-(dT) primer. cDNA synthesis was primed by the 3′ smRNA dT Primer, which incorporates an adapter sequence at the 5′ end of each first-strand cDNA molecule. In the template-switching step, PrimeScript RT uses the SMART smRNA Oligo as a template to add a second adapter sequence to the 3′ end of each first-strand cDNA molecule. Full-length Illumina adapters were added during PCR amplification. The forward PCR primer binds to the sequence added by the SMART smRNA Oligo, while the reverse PCR primer binds to the sequence added by the 3′ smRNA dT primer. The resulting library cDNA molecules had included sequences required for clustering on an Illumina flow cell. The libraries were validated by assessing their size, purity, and concentration using an Agilent Bioanalyzer. The libraries were pooled in equimolar amounts and sequenced on an Illumina HiSeq 2500 instrument (Illumina, San Diego, CA, USA) to generate 51 base reads. Image decomposition and quality value calculations were performed using the modules of the Illumina pipeline.

We uploaded the miRNAs that changed significantly after the 20 m SRT into miRDB (Liu and Wang 2019; Wong and Wang 2015), and chose genes scored above 80. To integrate functional genomic annotations, the Kyoto Encyclopedia of Genes and Genomes (KEGG) pathway in the Database for Annotation, Visualization, and Integrated Discovery (DAVID) was used (Dennis et al. 2003; Huang et al. 2009).

#### Statistical analysis

Statistical analysis was performed using GraphPad Prism ver. 7 software (Graph Pad Software Inc., La Jolla, CA, USA), and all diagrams and data are presented as mean ± standard deviation. Statistical significance was set at *p* < 0.05. Since one participant could not collect urine sample, the Kruskal–Wallis test was used to compare the data within the group. Spearman’s rank coefficient of correlation and simple linear regression were used to evaluate the relationship between the data.

## Results

### Effect of the 20 m shuttle run test (SRT) on exercise-related fatigue markers

**Fig. 2:**
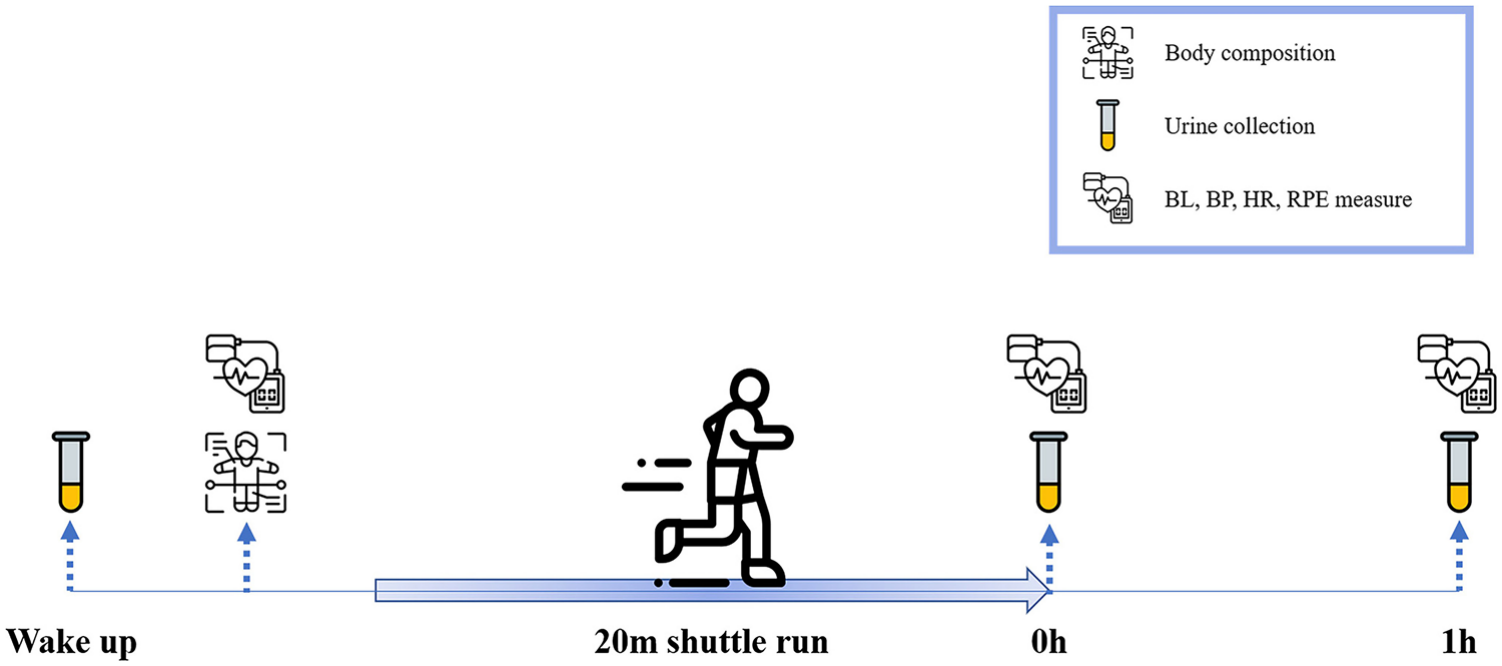
Workflow of the test day. Thirteen men participated in the 20 m SRT with their maximal efforts. Urine samples were collected and blood lactate (BLa), blood pressure (BP), heart rate (HR), and rating of perceived exertion (RPE) were measured before (Pre), after 0 h (Post), and after 1 h (Post 1 h) of the 20 m SRT

To verify whether the 20 m SRT could evoke fatigue to the participants, BLa, HR, RPE, and BP were measured before (Pre), immediately after (Post) the 20 m SRT, and after an hour of rest (Post 1 h) (Fig. 2). We observed significant changes in all fatigue markers after the 20 m SRT (Table 3, BLa, RPE, HR, and systolic blood pressure [SBP], *p* < 0.0001 and diastolic blood pressure [DBP], *p* < 0.01). All markers except for DBP (Table 3, *p* = 0.6435) were significantly elevated immediately after the 20 m SRT (Table 3, *p* < 0.0001). After 1 h of rest, BLa and RPE returned to normal state, HR was 7 ± 2 beat/min higher (*p* < 0.01), and SBP and DBP were 10 ± 3 mmHg (*p* < 0.05) and 6 ± 2 mmHg (*p* < 0.01) lower than the pre-exercise values, respectively (Table 3).

**Table 3:** Characteristics of commonly used fatigue markers after 20 m SRT

	Pre	Post	Post 1 h	*p*
BLa (mmol/L)	1.4 ± 0.3	11.4 ± 4	1.9 ± 0.6	< 0.0001
RPE	7 ± 1.5	17.5 ± 0.9	7.5 ± 1.2	< 0.0001
HR (beats/min)	65 ± 12	143 ± 22	72 ± 13	< 0.0001
SBP (mmHg)	120 ± 9	168 ± 25	110 ± 7	< 0.0001
DBP (mmHg)	74 ± 8	76 ± 11	68 ± 6	0.1199

### Characteristics of urinary extracellular vesicles (EVs) after the 20 m SRT

**Fig. 3:**
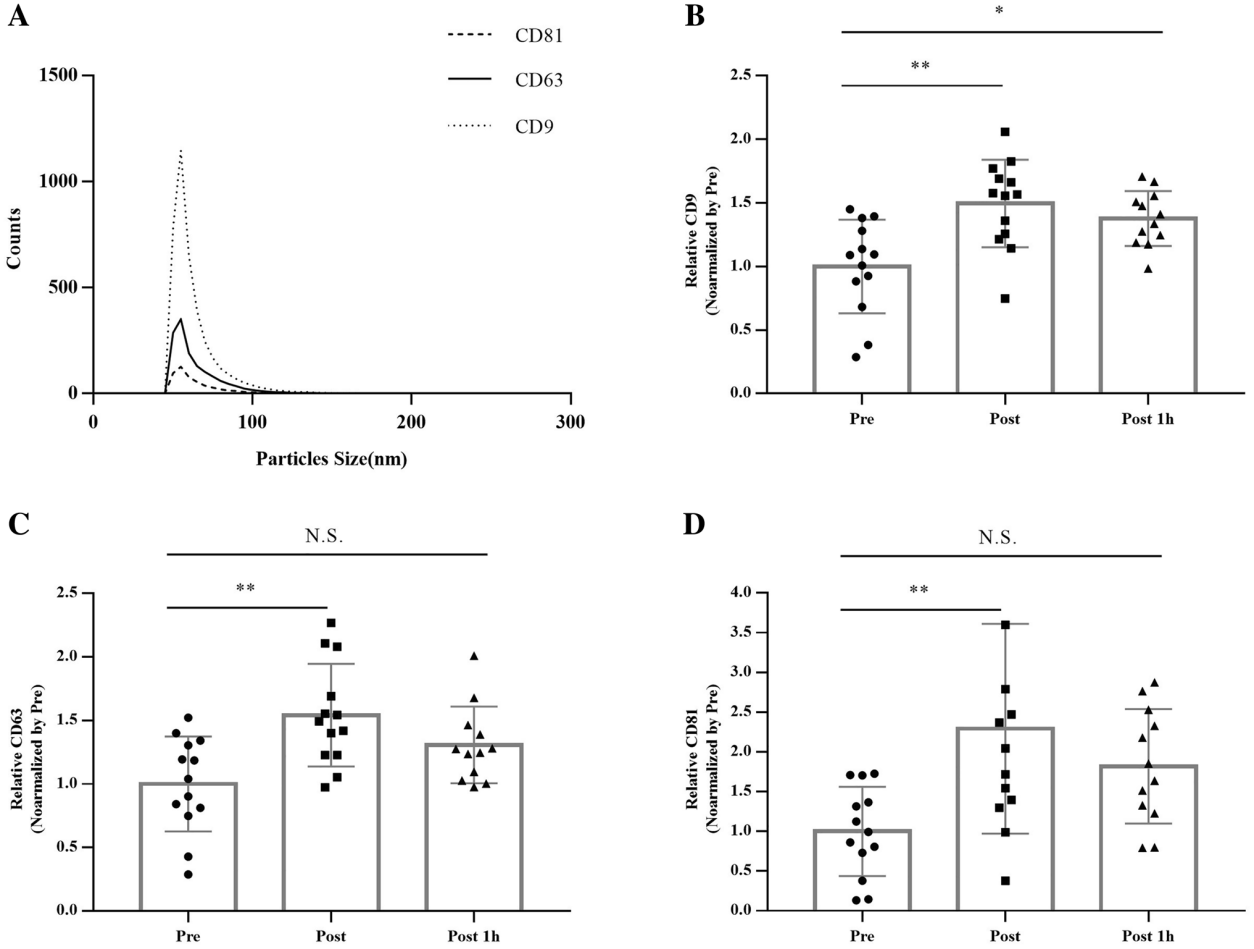
Characteristics of urinary EV marker proteins after the 20 m SRT. **a** The size distribution of all samples. (**b**–**d**) Relative concentrations of exosome marker proteins. Statistical analysis was performed with the aid of the ordinary one-way ANOVA with Tukey’s multiple comparisons test due to a missing sample (one participant could not collect the urine sample). The data represent means ± SEM. ^*^p < 0.05, ^**^p < 0.01, N.S. not significant

**Fig. 4:**
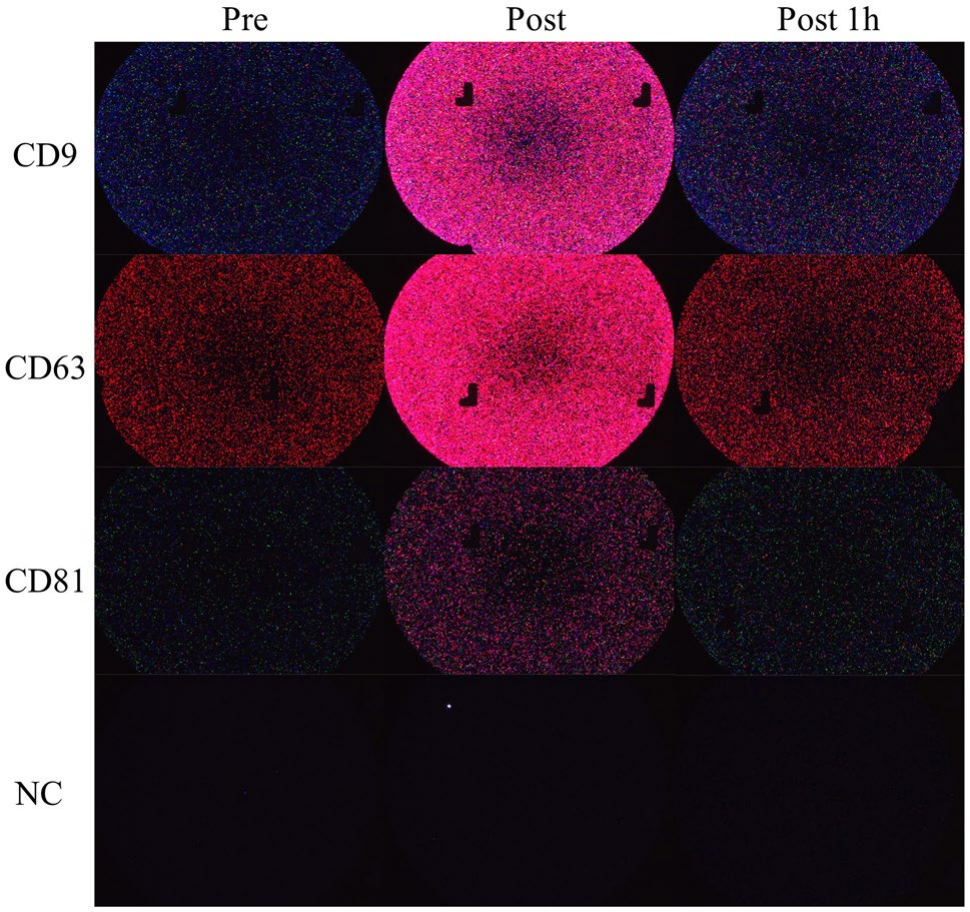
Representative images of captured urinary EV marker proteins (**a**–**d**). Captured EV marker proteins with anti-CD9 (**a**), anti-CD63 (**b**), anti-CD81 (**c**), and mouse IgG (**d**) on ExoView. Secondary labelling of exosomes captured on antibody capture spot: anti-CD 81/AF 555 (green), anti-CD 63/AF 647 (red) and anti-CD 9/AF 488 (blue)

To validate the characteristics of urinary EVs, particle size analysis was performed on the ExoView R-100 instrument (Figs. 3a and 4). Particles detected on the instrument were mostly 50 nm in size. The 20 m SRT significantly altered urinary EV marker proteins (Fig. 3b–d, *p* < 0.01). In particular, when the value was normalized by Pre, CD9, CD63, and CD81 in urine showed 1.5 ± 0.3, 1.5 ± 0.4, and 2.3 ± 1.3 higher concentrations in post-exercise samples than in the first morning urine (Pre), respectively (Fig. 3b–d, *p* < 0.01). After an hour of rest (Post 1 h), only CD9 levels were significantly higher than those in pre-exercise samples (Fig. 3b, *p* < 0.05).

### Correlation between urinary EVs and exercise-related fatigue markers

After identifying the characteristics of urinary EVs after the 20 m SRT, we analyzed the relationship between EV marker proteins in urine and exercise-related fatigue markers. When the data were treated as delta values (Post–Pre, Post 1 h–Post), significant correlations were found between urinary EVs and fatigue markers (Except for DBP, only CD81 showed a significant relationship Table 4).

**Table 4:** Correlation between urinary exosomal protein markers and BLa, RPE, HR, and BP

	Spearman r				
	Δ RPE	Δ HR	Δ SBP	Δ DBP	Δ BLa
**Δ** CD81	0.5342^**^	0.3128	0.4548^*^	0.2081	0.5061^**^
**Δ** CD63	0.6288^***^	0.5342^**^	0.5442^**^	0.3498	0.6854^***^
**Δ** CD9	0.5213^**^	0.4097^*^	0.5175^**^	0.1937	0.5308^**^

^*^, ^**^, ^***^ indicates significant differences between urinary EV marker proteins and fatigue markers at *p* < 0.05, 0.01, 0.001, respectively

### Urinary EV miRNA profiles after the 20 m SRT

**Fig. 5:**
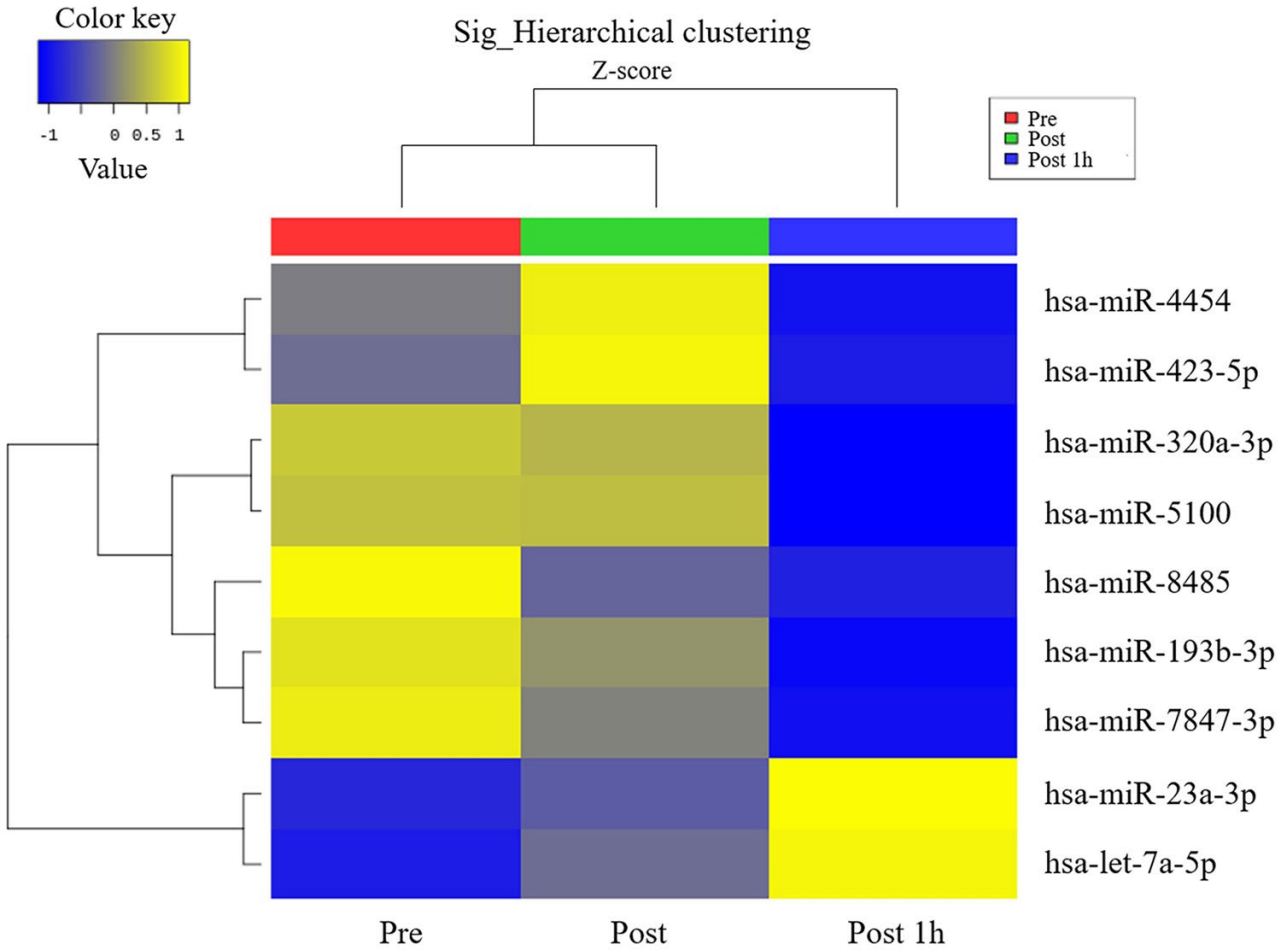
Effect of the 20 m SRT on urinary EV miRNA profiles. Microarray hierarchical clustering heat map image

Next, we performed miRNA sequencing of urinary EVs to identify the effects of exercise on EV components. Sequencing results showed a total of 9 upregulated and downregulated miRNAs after 20 m SRT (Fig. 5, Table 5).

**Table 5:**
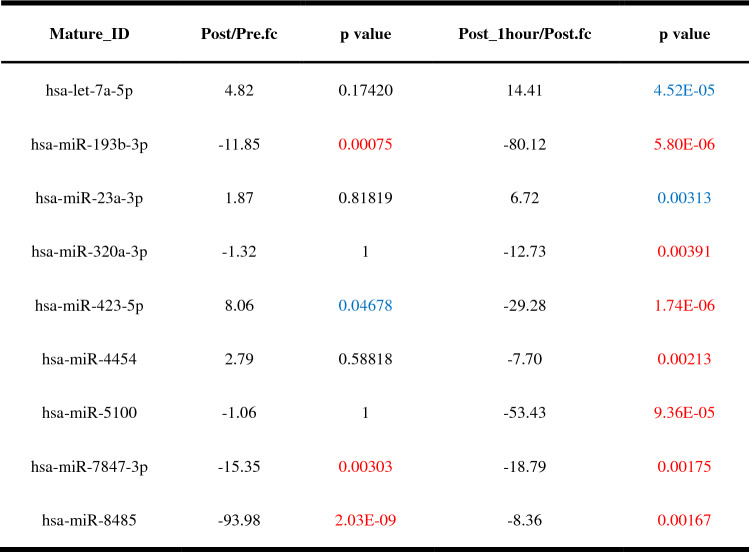
The list of changed miRNAs in urinary exosomes after 20 m SRT

Significantly upregulated and downregulated miRNAs at p < 0.05 are shown in blue and red, respectively

Since we found a significant increase in urinary EVs immediately after the 20 m SRT, we analyzed genes and possible pathways related to four miRNAs (hsa-miR-193b-3p, hsa-miR- 423-5p, hsa-miR-7847-3p, and hsa-miR-8485) showing significant alterations. The target genes of each miRNA were predicted by miRDB (http://mirdb.org/) and were functionally classified using the web-based gene functional annotation tool DAVID (https://david.ncifcrf.gov/home.jsp). Target genes of hsa-miR-193b-3p are presented in Table 6 (the number of target genes of hsa-miR-8485 was 1055, which is above the score of 80 on miRDB, data not shown). We could not find exercise-related genes in hsa-miR-423-5p and hsa-miR-7847-3p (data not shown). Interestingly, target genes of both hsa-miR-193b-3p and hsa-miR-8485 showed insulin-related pathways and terms (Tables 7 and 8).

**Table 6:** The list of target genes that regulated by has-miR-193b-3p (data from miRDB)

Target rank	Target score	Gene symbol	Target rank	Target score	Gene symbol	Target rank	Target score	Gene symbol	Target rank	Target score	Gene symbol
1	99	MAPK10	23	89	TAPT1	45	86	ATF7IP2	67	83	LRP4
2	98	PIGA	24	89	NT5E	46	86	ST6GALNAC5	68	83	ZNF618
3	97	DCAF7	25	89	PLAU	47	85	LAMC2	69	83	NAV3
4	97	RAPGEF6	26	89	TAOK1	48	85	KIT	70	83	ADGRE5
5	95	ERBB4	27	88	IGFBP5	49	85	KRAS	71	83	RBMXL1
6	95	ABI2	28	88	CYREN	50	85	RGMA	72	83	OSMR
7	94	DMXL2	29	88	CLTC	51	85	RUNX1T1	73	83	ADARB1
8	94	FLI1	30	88	HNRNPUL2	52	85	INO80D	74	82	CBX7
9	93	PLAG1	31	88	FAT4	53	85	AREL1	75	82	AAK1
10	93	VN1R1	32	88	CADM1	54	85	LAMC1	76	82	DNAAF4
11	93	SOS2	33	88	CNOT6	55	84	HOXD13	77	81	MSANTD2
12	92	KCNJ2	34	87	CCND1	56	84	TGFBR3	78	81	SRSF2
13	92	IL17RD	35	87	EPHA5	57	84	AIMP2	79	81	ZBTB25
14	92	FHDC1	36	87	CLOCK	58	84	YWHAZ	80	81	AJUBA
15	91	LYRM2	37	87	WDR82	59	84	UBP1	81	81	PLXNC1
16	91	CARF	38	87	AP2M1	60	84	P2RX5	82	80	ABCB8
17	90	ARHGEF12	39	87	GPR20	61	83	ZMAT3	83	80	NF2
18	90	MARF1	40	86	TSC1	62	83	CREBRF	84	80	USP53
19	90	RRAS2	41	86	CCDC28A	63	83	ETV1			
20	90	SLC10A6	42	86	ETS1	64	83	TIMM8B			
21	90	SIAH1	43	86	HEG1	65	83	PLCH1			
22	89	SLC16A6	44	86	UPRT	66	83	EARS2

**Table 7:** Functional annotation results of target genes regulated by has-miR-193b-3p

Category	Term	Genes	*p* value
KEGG_PATHWAY	hsa05221:Acute myeloid leukemia	CCND1, KIT, KRAS, SOS2, RUNX1T1	3.84E−04
KEGG_PATHWAY	hsa05200:Pathways in cancer	MAPK10, ARHGEF12, CCND1, KIT, KRAS, LAMC2, LAMC1, ETS1, SOS2, RUNX1T1	5.87E−04
KEGG_PATHWAY	hsa04151:PI3K-Akt signaling pathway	CCND1, KIT, TSC1, KRAS, LAMC2, LAMC1, OSMR, YWHAZ, SOS2	1.14E−03
KEGG_PATHWAY	hsa04917:Prolactin signaling pathway	MAPK10, CCND1, KRAS, SOS2	9.68E−03
KEGG_PATHWAY	hsa04014:Ras signaling pathway	MAPK10, KIT, RRAS2, KRAS, ETS1, SOS2	1.28E−02
KEGG_PATHWAY	hsa04012:ErbB signaling pathway	MAPK10, ERBB4, KRAS, SOS2	1.68E−02
KEGG_PATHWAY	hsa05231:Choline metabolism in cancer	MAPK10, TSC1, KRAS, SOS2	2.48E−02
KEGG_PATHWAY	hsa04510:Focal adhesion	MAPK10, CCND1, LAMC2, LAMC1, SOS2	3.88E−02
KEGG_PATHWAY	hsa04068:FoxO signaling pathway	MAPK10, CCND1, KRAS, SOS2	5.07E−02
KEGG_PATHWAY	hsa04910:Insulin signaling pathway	MAPK10, TSC1, KRAS, SOS2	5.45E−02
KEGG_PATHWAY	hsa04664:Fc epsilon RI signaling pathway	MAPK10, KRAS, SOS2	6.71E−02
KEGG_PATHWAY	hsa04010:MAPK signaling pathway	MAPK10, TAOK1, RRAS2, KRAS, SOS2	7.20E-02

**Table 8:** Functional annotation results of target genes regulated by has-miR-8485

Category	Term	Genes	*p* value
KEGG_PATHWAY	hsa04151:PI3K-Akt signaling pathway	ATF2, GSK3B, FLT1, IRS1, YWHAB, ITGB3, PTEN, FASLG, PIK3R1, EFNA5, IGF1R, BCL2L11, FGF9, YWHAQ, PPP2R1A, CD19, ITGB6, JAK2, PCK1, PDGFRA, CHUK, INSR, F2R, PRKCA, PPP2R5C, VEGFA, FGF14, CDK6, PPP2R2C, GNB4, COL6A3, GRB2, SGK1, EIF4E2, MET, FGFR3, CREB5	5.33E−05
KEGG_PATHWAY	hsa04010:MAPK signaling pathway	ATF2, SRF, CACNA1B, FASLG, NLK, STK3, RASGRP3, CRKL, ELK4, CACNG8, DUSP10, FGF9, GNA12, MAP3K2, PDGFRA, TGFB2, JUND, CHUK, PLA2G4A, PRKCA, MAPK14, DUSP6, CACNB2, PPM1B, FGF14, MAPKAPK2, GRB2, MAPT, LAMTOR3, FGFR3	5.49E−05
KEGG_PATHWAY	hsa05200:Pathways in cancer	GSK3B, CTBP2, HHIP, PTEN, FASLG, PIK3R1, CBL, ETS1, FOXO1, GNAI1, IGF1R, RASGRP3, CRKL, EDNRA, FGF9, GNA12, E2F1, EP300, E2F3, SMAD2, STAT5A, PDGFRA, STAT5B, TGFB2, TCF7L1, CHUK, PTCH1, F2R, ARNT, PRKCA, RUNX1, VEGFA, BMP2, FGF14, CDK6, GNB4, CCDC6, GRB2, MET, FGFR3	7.90E−05
KEGG_PATHWAY	hsa05161:Hepatitis B	STAT5A, ATF2, STAT5B, TGFB2, EGR3, CHUK, YWHAB, SRC, PTEN, FASLG, PRKCA, PIK3R1, DDB1, CDK6, YWHAQ, E2F1, EP300, GRB2, E2F3, CREB5	1.95E−04
KEGG_PATHWAY	hsa04068:FoxO signaling pathway	SMAD2, TGFB2, USP7, CHUK, IRS1, INSR, PTEN, FASLG, CSNK1E, PIK3R1, NLK, MAPK14, FOXO1, IGF1R, BCL2L11, EP300, GRB2, SGK1, PCK1	2.09E−04
KEGG_PATHWAY	hsa04360:Axon guidance	EPHA5, GSK3B, EPHA4, UNC5A, UNC5B, SEMA6D, SEMA4C, UNC5C, EFNA5, GNAI1, ABLIM1, DPYSL2, PAK6, PLXNC1, SRGAP3, PAK3, MET, EPHB3	3.26E−04
KEGG_PATHWAY	hsa05220:Chronic myeloid leukemia	STAT5A, STAT5B, TGFB2, CTBP2, CHUK, PIK3R1, CBL, CRKL, RUNX1, CDK6, E2F1, GRB2, E2F3	3.29E−04
KEGG_PATHWAY	hsa04810:Regulation of actin cytoskeleton	CYFIP2, PDGFRA, CYFIP1, ITGAM, GSN, SRC, ITGB3, F2R, RDX, PIK3R1, SSH2, FGD1, SSH1, CRKL, MYLK, FGF14, FGF9, GNA12, PIP4K2A, ARHGEF4, PAK6, ITGB6, PAK3, FGFR3	6.27E−04

## Discussion

In the present study, urinary EVs showed higher levels after the 20 m SRT. Moreover, the change in urinary EVs was related to the commonly used fatigue markers. Another key finding of this study was that acute exercise altered urinary EV miRNAs including has-let-7a-5p, hsa-miR-193b-3p, hsa-miR-23a-3p, hsa-miR-320a-3p, hsa-miR-423-5p, hsa-miR-4454, hsa-miR-5100, hsa-miR-7847-3p, and hsa-miR-8485. It has been well documented that exercise induces the secretion of EVs in circulation (Safdar et al. 2016; Siqueira et al. 2021); however, alterations in urinary EVs after physical exercise have not been explored. To the best of our knowledge, the present study is the first to demonstrate that exercise induces changes the number of urinary EVs.

Although numerous studies support the beneficial effect of exercise in preventing chronic diseases and early death (Penedo and Dahn 2005; Viña et al. 2016), excessive exercise could result in fatigue (Cordeiro et al. 2017) that reduces exercise capacity (De Becker et al. 2000). The commonly used markers of exercise-induced fatigue or internal intensity are BLa (Finsterer 2012), RPE (Falk Neto et al. 2020), HR (Berglund et al. 2019), and BP (Sharman and Lagerche 2015). In this study, we first examined whether the 20 m SRT could evoke fatigue in study participants. A previous study suggested that 20 m SRT predicts VO_2max_ and induces peak BLa and HR as high as the incremental exercise test on a treadmill (Aandstad et al. 2011). Herein, significantly elevated levels of BLa, HR, RPE, and SBP in each individual after the 20 m SRT support the evidence that the 20 m SRT could elicit exhaustion (Ahmaidi et al. 1992). In accordance with a previous study, DBP did not show significant changes after the 20 m SRT (Miyai et al. 2002). However, variables that were measured at the post time point may not represent the peak values, since the participants took at least 30 s to reach to the measurement spot (Pescatello et al. 1991). Nevertheless, overall, we conclude that 20 m SRT could evoke exercise-induced fatigue to the participants.

EVs are naturally secreted from cells and are bound to lipid bilayers containing proteins, lipids, mRNA, non-coding RNA, and miRNA (Linxweiler and Junker 2020). After release, exosomes transfer bioactive molecules to recipient cells and are highlighted as key regulators in cell-to-cell communication (Wortzel et al. 2019). In recent studies, EVs have been presented as potentially novel biomarkers of diverse diseases and disorders (Mussack et al. 2019; Pitt et al. 2016; Shibata 2021; Yang et al. 2020).

Previous studies have suggested that the number of EV particles in circulation is elevated immediately after exercise bout both in humans (Frühbeis et al. 2015b; Whitham et al. 2018) and animals (Oliveira et al. 2018). One study demonstrated that the concentrations of CD9, CD63, and CD81 in plasma EVS were increased after exercise with exhaustion, but the particle counts did not change (Brahmer et al. 2019). The present data describing the characteristics of urinary EVs after a single bout of exercise until voluntary exhaustion suggests that EV particles were abundant in the urine after exercise. Here, we used the ExoView platform (Nanoview Bioscience, Boston, USA), which captures CD9, CD63, and CD81-positive EV particles on a single-vesicle basis, to assess the characteristics of urinary EVs (Bachurski et al. 2019). Since the platform identifies each particle that is colored with respective antibodies (CD9, CD63, and CD81), it could detect EVs in urine precisely (Bachurski et al. 2019). We had found all EV marker protein-positive particles were elevated in urine, which coincides with that of plasma EVs after cycling exercise until exhaustion (Brahmer et al. 2019; Whitham et al. 2018). However, since circulating EVs have been reported to be elevated, not only late but also early in high intensity exercise when participants may not feel fatigue, more time points of measuring urinary EVs is needed to elucidate the relationship between urinary EVs and fatigue.

Next, we determined if the quantitative changes in urinary EVs (CD9-, CD63-, and CD81-positive particles) correlated with RPE, HR, SBP, and BLa. Urinary EVs were found to be associated with commonly used exercise fatigue or intensity markers. In line with our findings, previous studies have reported that exosome secretion could be facilitated by various acute stressors (Beninson and Fleshner 2014) and that elevated plasma exosome concentration could be observed in patients with chronic fatigue syndrome (Castro-Marrero et al. 2018). CD81 can be upregulated by oxidative stress, enhancing monocyte adhesion to endothelium under flow (Rohlena et al. 2009). EVs could manage reciprocal communication between heart and other organs (Gabisonia et al. 2022) and take part in the development of various hypertensive disorder(Z. Z. Liu et al. 2021). Consequently, we suggest that urinary EVs might reflect the acute fatigue state.

Since exercise modifies the urinary proteome (Kohler et al. 2015) and EV miRNA profile in plasma (Yoon et al. 2021), and both urine and urinary EVs comprise the plasma proteome (Dear et al. 2013; Harpole et al. 2016), we hypothesized that exercise could change the urinary EV miRNA contents. After 20 m SRT and 1 h of rest, a total of 9 miRNAs in urinary EVs were found to be altered. Among them, five miRNAs (hsa-let-7a-5p. hsa-miR-193b-3p, hsa-miR-23a-3p, hsa-miR-320a-3p, and hsa-miR-423-5p) were highly abundant in human urinary EVs, supporting our data (Cheng et al. 2014). Another previous study showed that hsa-miR-5100 was found only in urine while hsa-let-7a-5p, hsa-miR-23a-3p, hsa-miR-320a-3p, hsa-miR-423-5p, hsa-miR-7847-3p, hsa-miR-4454, and hsa-miR-8485 were common in both urine and serum (Park et al. 2020). A pilot study conducted in 2021 revealed that hsa-miR-23a-3p was elevated in urinary as well as in plasma EVs (Kuji et al. 2021), which is similar to our finding that hsa-miR-23a-3p gradually increased with time. After intensive exercise, circulatory hsa-miR-23a-3p was upregulated in plasma EVs, but downregulated in the muscles (D’souza et al. 2018).

Next, using an online database for miRNA target prediction, we identified 84 genes and 1055 genes that were regulated by hsa-miR-193b-3p and hsa-miR-8485, respectively. Hsa-miR-423-3p targeted only 3 genes, while hsa-miR-7847-3p targeted 74 genes (data not shown). Then, using DAVID, which is a Web-based functional genomic annotation tool, we verified whether these genes could mediate exercise response. Interestingly, hsa-miR-193b-3p target genes might functionally modulate the PI3K-Akt, insulin, and mitogen-activated protein kinase (MAPK) pathways. A similar result was obtained for the hsa-miR-8485 target genes. Given the beneficial effects of exercise (Prior et al. 2004) and the possible function of EVs in cell-to-cell communication (Mathieu et al. 2019), it is possible that altered urinary EV miRNAs are involved in insulin sensitivity by direct interaction with organs responding to insulin (Kumar et al. 2021). Normally, exercise until exhaustion can induce a small hyperglycemic response and persists for up to 1 h, and this is accompanied by hyperinsulinemia in recovery (Marliss and Vranic 2002). In addition, a single bout of exercise is reported to increase insulin sensitivity for at least 16 h (Borghouts and Keizer 2000). In this study, hsa-miR-193b-3p and hsa-miR-8485 were downregulated 1 h after 20 m SRT.

Exercise stimulation increases the activity of MAPK, which is involved in the phosphorylation of substrates related to carbohydrate, fat metabolism, and hypertrophy (Kramer and Goodyear 2007). In accordance with this, secreted extracellular vesicles after exercise showed upregulated glycolytic proteins, suggesting that circulating EVs modulate crosstalk between tissues 60. In addition, a previous study has reported that exercise affects growth hormone and insulin-like growth factor-1 (IGF-1) and PI3K/AKT pathways (Moon et al. 2020), resulting in cardiac hypertrophy and protection (Weeks et al. 2017). Thus, EVs may be involved in the regulation of energy metabolism (de Mendonça et al. 2020; Göran Ronquist 2019).

There are some limitations to the present study. First, the small sample size and characteristics of the participants, as the effects of sex differences and aging on circulating EVs have been reported (Bertoldi et al. 2018; Jayachandran et al. 2015). Second, the measurement of HR, BP, and BLa was delayed by approximately 30 s since the participants had to come to the measuring spot. Third, we did not control the water consumption among the participants. Fourth, the present study proceeded without a control group. Further study is needed, including appropriate control (rest only or mild intensity exercise) with more measurement time points. Finally, for miRNA analysis, we pooled EV samples into one sample due to the small volume of total yield.

Despite these limitations, this study is the first to demonstrate urinary EVs and their contents after exercise. We found a significant increase in urinary EVs after 20 m SRT, and this increase subsided after 1 h of rest. The variation in urinary EVs showed a significant correlation with common exercise fatigue or internal intensity markers. In addition, we identified changes in urinary EV miRNAs in urine, their target genes, as well as possible biological functions that could be mediated by altered genes. These results confirmed that urinary EVs could be potential biomarkers of exercise-induced fatigue.

## Conclusion

In conclusion, acute exercise until voluntary exhaustion changed the concentration of urinary EVs and miRNA profiles. In addition, downregulated miRNAs, hsa-miR-193b-3p and hsa-miR-8485, in urinary EVs were identified to mediate energy metabolism-related pathways. Therefore, this study suggests that urinary EVs are potential biomarkers of fatigue after a single exercise session in young adult males.

## Data Availability

The datasets that support the findings of this study are openly available in online repositories. The names of the repository/repositories and accession number(s) can be found at: https://www.ncbi.nlm.nih.gov/geo/query/acc.cgi?acc=GSE193186.

## Abbreviations

EV: Extracellular vesicle
miRNA: MicroRNA
BLa: Blood lactate
HR: Heart rate
RPE: Rating of perceived exertion
SBP: Systolic blood pressure
DBP: Diastolic blood pressure
MVB: Multivesicular body
SRT: Shuttle run test
